# Impact of the circadian nuclear receptor REV-ERBα in dorsal raphe 5-HT neurons on social interaction behavior, especially social preference

**DOI:** 10.1038/s12276-023-01052-7

**Published:** 2023-08-03

**Authors:** Sangwon Jang, Inah Park, Mijung Choi, Jihoon Kim, Seungeun Yeo, Sung-Oh Huh, Ji-Woong Choi, Cheil Moon, Han Kyoung Choe, Youngshik Choe, Kyungjin Kim

**Affiliations:** 1 Department of Brain Sciences, Daegu Gyeongbuk Institute of Science and Technology (DGIST), Daegu, 42988 Republic of Korea; 2 Convergence Research Advanced Centre for Olfaction, Daegu Gyeongbuk Institute of Science and Technology (DGIST), Daegu, 42988 Republic of Korea; 3 Korea Brain Research Institute (KBRI), Daegu, 41062 Republic of Korea; 4 Department of Pharmacology, College of Medicine, Institute of Natural Medicine, Hallym University, Chuncheon, 24252 Republic of Korea; 5 Department of Electrical Engineering and Computer Science, Daegu Gyeongbuk Institute of Science and Technology (DGIST), Daegu, 42988 Republic of Korea

**Keywords:** Circadian regulation, Molecular neuroscience

## Abstract

Social interaction among conspecifics is essential for maintaining adaptive, cooperative, and social behaviors, along with survival among mammals. The 5-hydroxytryptamine (5-HT) neuronal system is an important neurotransmitter system for regulating social behaviors; however, the circadian role of 5-HT in social interaction behaviors is unclear. To investigate whether the circadian nuclear receptor REV-ERBα, a transcriptional repressor of the rate-limiting enzyme tryptophan hydroxylase 2 *(Tph2)* gene in 5-HT biosynthesis, may affect social interaction behaviors, we generated a conditional knockout (cKO) mouse by targeting *Rev-Erbα* in dorsal raphe (DR) 5-HT neurons (5-HT^DR^-specific REV-ERBα cKO) using the CRISPR/Cas9 gene editing system and assayed social behaviors, including social preference and social recognition, with a three-chamber social interaction test at two circadian time (CT) points, i.e., at dawn (CT00) and dusk (CT12). The genetic ablation of *Rev-Erbα* in DR 5-HTergic neurons caused impaired social interaction behaviors, particularly social preference but not social recognition, with no difference between the two CT points. This deficit of social preference induced by *Rev-Erbα* in 5-HT^DR^-specific mice is functionally associated with real-time elevated neuron activity and 5-HT levels at dusk, as determined by fiber-photometry imaging sensors. Moreover, optogenetic inhibition of DR to nucleus accumbens (NAc) 5-HTergic circuit restored the impairment of social preference in 5-HT^DR^-specific REV-ERBα cKO mice. These results suggest the significance of the circadian regulation of 5-HT levels by REV-ERBα in regulating social interaction behaviors.

## Animal behavior: Nighttime decrease in serotonin important for social interaction

Daily fluctuation of serotonin levels during the day, controlled by a nuclear receptor called REV-ERBα, is important in social behavior and may offer insights into conditions such as autism spectrum disorder (ASD). The neurotransmitter serotonin influences behavior and mood, but how its circadian fluctuations affect social behavior is not fully understood. Kyungjin Kim at the Daegu Gyeongbuk Institute of Science & Technology in Daegu, South Korea, and co-workers used mice lacking REV-ERBα to investigate links between brain chemistry and social behaviors. These mice, in contrast to controls, showed no preference for a novel mouse over an empty cage. Real-time measurement linked this behavior to higher nighttime levels of serotonin in the brains of the receptor-deficient mice. These results contribute to understanding the biology underlying social behavior and may help identify interventions for conditions such as ASD.

## Introduction

5-Hydroxytryptamine (5-HT, serotonin) has various physiological and behavioral functions, including the regulation of mood, the sleep-wake cycle, cognition, memory, reward, and sociability^1^. It is synthesized in a stepwise manner from the amino acid tryptophan by two enzymes, tryptophan hydroxylase (TPH) and aromatic amino acid decarboxylase (AADC). In mammals, there are two TPH isoforms (encoded by *Tph1* and *Tph2*); TPH2 is mainly expressed in the central nervous system (CNS), whereas TPH1 regulates 5-HT synthesis in peripheral organs such as the duodenum^2^. Moreover, 5-HT neurons are mostly localized in the dorsal and medial parts of the raphe nucleus (DR and MR) in the brainstem and innervate various cortical or subcortical regions, including the prefrontal cortex (PFC), nucleus accumbens (NAc), ventral tegmental area (VTA), hippocampus and other regions^3^.

5-HTergic neuronal circuits are essential for regulating social interaction behaviors. Within the raphe nucleus, neuronal subpopulations regulating social preference and social recognition appear functionally and anatomically distinct^4,5^. For instance, DR 5-HTergic innervation to the NAc is crucial for social preference^6^, while MR 5-HTergic neurons innervate the medial septum (MS) and regulate social memory via the MS-dorsal CA2 of the hippocampal circuit^7^. In addition, many other studies suggest that dysfunction of the 5-HT system causes abnormal social interaction behaviors, notably autism spectrum disorder (ASD)^8,9^^,^ and that increased presynaptic serotonin activity and serotonin levels are related to social behavior in humans^10^^,^ but the underlying cellular mechanisms are largely unknown.

In rodents, 5-HT levels in the CNS exhibit circadian rhythm^11,12^. This means that 5-HT may be influenced by the circadian time-keeping system. Briefly, the circadian time-keeping system consists of two interlocking transcriptional/translational negative feedback loops that represent the core and stabilizing (auxiliary) loops^13^. In the core loop, the transcriptional activators CLOCK (Circadian Locomotor Output Cycles Kaput) and BMAL1 (Brain and Muscle ARNT-Like1) form a heterodimer and then activate E-box-mediated transcription of downstream genes, such as *Periods* (*Per1/2*), *Cryptochromes* (*Cry1/2*), and clock-controlled genes. In the stabilizing loop, CLOCK and BMAL1 heterodimers regulate the expression of circadian nuclear receptor genes, such as *Rorα* and *Rev-erbα*. REV-ERBα (encoded by *NR1D1*) is a nuclear receptor subfamily 1 protein and serves as a transcriptional repressor modulating *Bmal1* gene expression by competitive binding with ROR*α* and REV-ERBα in the Bmal1 promoter^14^. We previously found that REV-ERBα repressed thyroxine hydroxylase (*TH*) gene transcription via competition with NURR1, another nuclear receptor-related 1 protein in the dopaminergic neurons of the ventral tegmental area (VTA), thereby driving circadian expression of the *TH* gene. Furthermore, we disclosed a molecular link between the circadian timing-keeping system and mood-related behaviors^15^. A recent study using a short-hairpin RNA-mediated gene knockdown technology showed that decreased REV-ERBα expression in the NAc affected social and mood-related behaviors^16^, suggesting the possible involvement of REV-ERBα activity in deficits in social interaction behaviors; however, the underlying neural mechanism is unclear. We attempted to explore whether the circadian nuclear receptor REV-ERBα in DR 5-HT neurons has a functional link with social interaction behaviors.

In the present study, we genetically ablated the *Rev-Erbα* gene in DR 5-HTergic neurons using an adeno-associated viral (AAV)-mediated CRISPR/Cas9 system, producing 5-HT^DR^-specific REV-ERBα conditional knockout (cKO) mice, and assayed social interaction behaviors with a three-chamber social interaction test at two circadian time (CT) points, CT00 and CT12. We also attempted to correlate behavioral alterations with real-time 5-HT neuron activity and 5-HT levels in the DR and NAc using two imaging sensors, GCaMP7s and iSeroSnFR, with the fiber-photometric device. Finally, optogenetic manipulation of DR 5-HTergic neuron to NAc circuit rescued the deficit in social preference in DR 5-HT REV-ERBα cKO mice.

## Materials and methods

### Animals

Male C57BL/6 J, SERT-cre (Slc6a4-cre, JAX:014554), and Rosa26-LSL-Cas9 (JAX:026179) (8–15 weeks old) mice were used as experimental subjects. All animals used in this study were housed in a temperature-controlled (23–25 °C) environment under a 12/12 h light/dark (LD) photoperiod (lights on at 7:00 AM) with *ad libitum* access to food and water. For circadian time (CT) course experiments, animals were constantly kept in the dark for a minimum of 3 days and then assigned to social behavioral tests at two CT points, CT22-01 (CT00) vs. CT10-13 (CT12), under dim red light. All animal procedures were approved by the Institutional Animal Care and Use Committee of DGIST.

### Molecular cloning adeno-associated virus (AAV) vectors and virus production

#### Single-guide RNA (sgRNA)

sgRNA targeting *Rev-erbα* was designed and selected using CHOPCHOP^17^. The synthesized DNA oligomers with PAM adaptor sequences were annealed and cloned into the cre-dependent sgRNA expression vector expressing HA (hemagglutinin), AAV2/PHP.eB-CMV-FLEX-SaCas9-U6-sgRNA^18^. sgRNA sequences were designed as follows: sgRev-erbα, 5'-GTT GCG ATT GAT GCG AAC GAT GG-3' (chr 11: 98,771,255, strand: +).

#### AAV production

All recombinant AAV vectors used in this study were generated as described in a previous study^19^ with minor modifications. After AAV production, the titer was quantified using real-time qPCR. AAV vectors were injected into the DR or NAc of 8-week-old mice and used at the following titers and volumes: AAV2/PHP.eB-CMV-FLEX-SaCas9-U6-Rev-erbα-sgRNA, 1.0 × 10^13^ GC/ml; 800 nl; AAV/PHP.eB-DIO-GCaMP7s-WPRE^20^, 1.0 × 10^13^ GC/ml; 800 nl; AAV-PHP.eB-CAG-iSeroSnFR-Nlgn^21^, 1.0 × 10^13^ GC/ml; 800 nl; AAV2 retro-U6-Rev-erba-sgRNA-hSyn-mCherry, 1.13 × 10^14^ GC/ml; 500 µl; AAV2/PHP.eB-EF1a-DIO-CHR2 (C128S/D156A)-mCherry (SSFO)^22^, 1.0 × 10^13^ GC/ml; 800 nl AAV1-EF1a-DIO-NpHR3.0-eYFP-WPRE-hGH^23^, 1.0 × 10^13^ GC/ml; 500 nl.

#### Surgical procedures

Mice were deeply anesthetized with an intraperitoneal injection of pentobarbital sodium (50 mg/kg) and placed on a stereotaxic apparatus (Stoelting). The coordinates were lambda AP, +1.5 mm ML, −3.0 mm DV, angled 20° for unilateral AAV injection and cannula implantation into the DR and +1.5 mm AP, ±0.5 mm ML, −3.2 mm DV; bilateral AAV injection and optic fiber implantations above the NAc, respectively.

### Drug preparation and application

#### Local microinjection of SR8278 or GSK4112

The REV-ERBα antagonist SR8278 (Tocris Bioscience) was dissolved in ethanol to a concentration of 50 μg/μL, as described in a previous study^24^, and the REV-ERBα agonist GSK4112 (Sigma‒Aldrich) was dissolved in DMSO (Sigma‒Aldrich) to a concentration of 100 μM. SR8278 (16 μg/mouse) and GSK4112 (32 ng) were directly microinjected using a 26-gauge cannula (PlasticOne) into the DR with a Hamilton syringe at a rate of 0.1 μl/min 3 h before social interaction tests.

### RNA isolation and real-time qPCR

Before euthanizing the mice for tissue sampling, avertin (300 mg/kg) was administered via intraperitoneal injection. RNA samples were purified using TRIzol reagent (Invitrogen) according to the manufacturer’s protocol. For RT‒PCR, 1 μg of each RNA sample was reverse-transcribed using the PrimeScript RT reagent (Takara Bio). Aliquots of the cDNA were then subjected to real-time qPCR in the presence of SYBR Green I (Enzynomics). Gene expression levels were normalized to the gene encoding TATA-box binding protein (Tbp).

### High-performance liquid chromatography-coupled electrochemical detector (HPLC-ECD)

#### Sample preparation

5-HT levels were measured in the DR and NAc regions. The mouse brain was dissected (2 mm thickness) using a brain matrix. The rest of the brain regions were removed manually using a blade, and the DR and NAc regions were micropunched (2 mm diameter) and stored at −80 °C until further processing.

#### Measurements of 5-HT and its metabolites

Brain tissues were washed with prechilled phosphate-buffered saline and homogenized using a sonicator with pulses at 12 volts in 0.3 N perchloric acid solution. For HPLC-ECD analysis, the cell debris was pelleted via centrifugation at 14,000 × *g* for 20 min at 4 °C using a refrigerated centrifuge (Eppendorf). The level of 5-HT was measured by HPLC-ECD (HTEC-510; Eicom) using a SC-5ODS column (2.1ɸ); 20 μl of supernatant was injected into the HPLC-ECD. The mobile phase was prepared using 100 mM citrate-acetate buffer (pH 3.9), methanol (83:17, v/v), 140 mg/l Na-octane sulfonate, and 5 mg/l EDTA 2Na, according to the manufacturer’s instructions. HPLC-grade reagents were purchased from Sigma‒Aldrich.

### Three-chamber social interaction test

A three-chamber sociability assay was performed in an arena with three separate chambers^25,26^. The subject mouse was habituated to the arena, with two empty cups placed in the two outer chambers for 10 min. Afterward, a young C57BL/6 J WT mouse (6–8-week-old male) was placed in a cup in the right chamber as a cup containing a novel mouse, and the subject mouse was placed in the middle chamber for 5 min. The barriers were then raised, and the subject mouse was allowed to explore freely for 10 min for the social preference test. Afterward, in the social recognition test, another novel mouse was introduced into the empty cup on the left side and the subject mouse was allowed to explore the other two chambers freely for 10 min. The investigation time was quantified automatically using a video tracking system (Ethovision). The social preference index was calculated by the following formula: [investigation time with a novel mouse – investigation time with empty cup]/[investigation time with novel mouse + investigation time with empty cup].

### Fiber photometry

Following AAV-DIO-GCaMP7s or AAV-CAG-iSeroSnFR virus injection, an optical fiber (230 μm O.D., 0.37 numerical aperture (NA)) was placed in a ceramic ferrule and inserted toward the DR through stereotactic surgery. The optical fiber and skull were fixed together using a screw and dental cement. Mice were individually housed for at least two weeks for recovery. To avoid autofluorescence of the patch, cable bleaching was performed by exposing the patch cable (Doriclenses) to 500 mA light for >1 h before the experiments. We used an optical power meter (Thorlabs) and adjusted the light intensity to <30 µW at the end of the fiber optic cable during all recordings. All recordings were performed using TDT synapse software (Tucker-Davis Technologies) through GCaMP7s or iSeroSnFR excitation at two wavelengths (465 nm calcium or 5-HT-dependent signal and 405 nm isosbestic control). After recording fluorescence signals (GCaMP7s or iSeroSnFR) of freely moving mice within 60 min at 6-h intervals, all data were fitted by a custom-written MATLAB script. All signal changes in fluorescence were calculated over baseline fluorescence as $\triangle F/F=F(t)-F(0)/F(0)$ (F(0): fitted 405 nm signal, F(t): calcium- or iSeroSnFR-dependent 470 nm signal) to calculate normalized ∆F/F^27^. Then, peak detection was set at the habituation peak amplitude average, and the number of detected peaks per min was divided by 60 to indicate the number of peaks per min at each time point. For fluorescence recording of GCaMP7s and iSeroSnFR during social interaction, the signal was only considered if the mouse was in the arena of social interaction for the first approach over 10 seconds with video recording.

### Optogenetic manipulation

For optogenetic stimulation experiments, 800 nl of AAV expressing cre-dependent stabilized step-function opsin (SSFO)^22^ or NpHR 3.0^22^ was unilaterally injected into the DR, and a bilateral fiber optic cannula was implanted (200 μm diameter, 0.5 NA; Neurophotometrics). To stimulate the 5-HTegic axon terminals of cre-dependent SSFO-expressing neurons, a single 8 s light pulse with 3.8 mW of blue light (28–30 mW/mm^2^ at the tip of the patch cords) generated using a 473 nm laser was delivered to the mouse bilaterally. For NpHR 3.0 stimulation, 15 mW of the 532 nm laser was delivered to mice bilaterally (200 μm diameter, 0.5 NA; Neurophotometrics). The mouse received cycles of 8 s light on and 2 s light off for NpHR stimulation.

### Immunohistochemistry

For histological analysis, animals were perfused with freshly made 4% paraformaldehyde in phosphate buffered saline (PBS). Brain samples were postfixed in the same fixative overnight at 4 °C. Then, tissues were cryoprotected in 30% sucrose and sectioned (20 µm thickness) using a cryostat. For TPH2 and REV-ERBα labeling, sections were incubated with 0.3% PBST containing 10% goat serum at RT for 2 h for blocking and then incubated with primary antibodies against TPH2 (1:1000, Abcam), HA (1:1000), and REV-ERBα (1:200, Abcam) overnight at 4 °C. After washing with 0.1% PBST, a secondary antibody was applied for 2 h; sections were then mounted and observed under a confocal microscope (Zeiss LSM700 and 800).

### Statistical analysis

Statistical analyses were performed using Prism8 (GraphPad). We performed two-way ANOVA followed by Tukey’s post hoc test for RT‒qPCR, HPCL-ECD, and social behavior tests and Sidak’s test for fiber-photometry and optogenetic experiments. Data are presented as the mean ± s.e.m. Significance was defined as *p* < 0.05. To minimize observer bias in data analysis, the observers were randomly assigned video recordings, and blind data analyses were performed.

## Results

### 5-HT^DR^-specific REV-ERBα cKO mice exhibited altered circadian rhythms of Tph2 and 5-HT levels

**Fig. 1:**
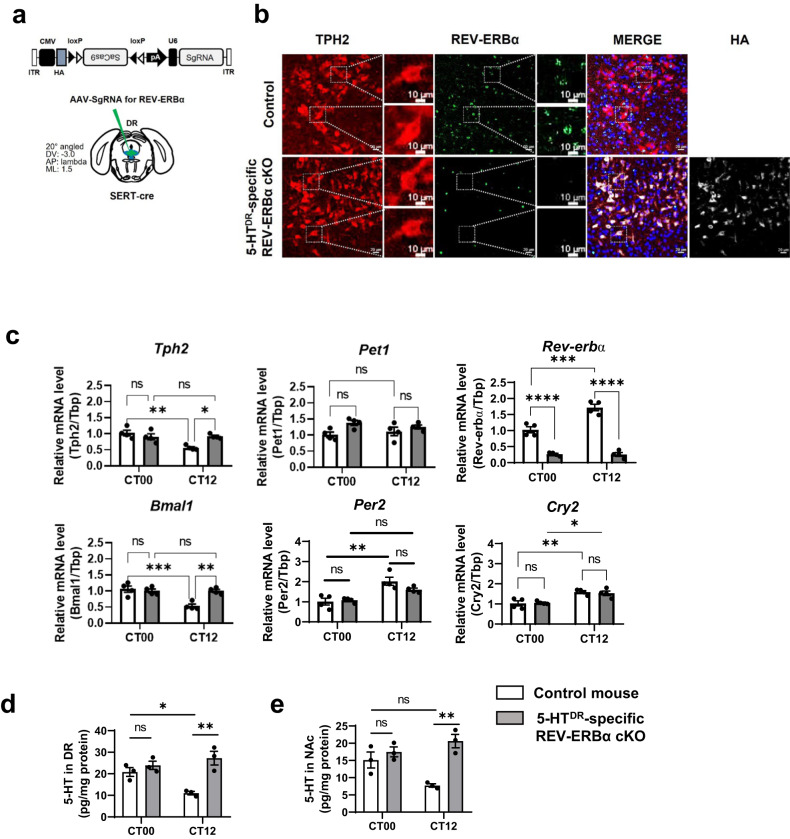
5-HT^DR^-specific REV-ERBα cKO mice exhibit alterations in daily mRNA expression and 5-HT levels in the DR. **a** Design of AAV-PHP.eB-FLEX-SaCas9-U6-sgRNA for 5-HT^DR^-specific REV-ERBα cKO and stereotactic injection site into the DR of control (SERT-cre) mice. **b** Histological image of DR in control and 5-HT^DR^-specific REV-ERBα cKO mice. The scale bar indicates 20 μm. The right upper boxes represent a high-magnification image of a white arrow. The scale bar indicates 10 μm. **c** mRNA expression profiles of 5-HT-related genes (*Tph2*, *Pet-1*, and *Rev-erbα*) and circadian clock genes (*Bmal1*, *Per2*, and *Cry2*) in the DR were examined at two circadian time points, CT00 and CT12, in control and 5-HT^DR^-specific REV-ERBα cKO mice using real-time qPCR. mRNA levels were normalized using the expression level of a housekeeping gene, TATA-box-binding protein (Tbp). **d** The amount of 5-HT in the DR was quantified at CT00 and CT12 in the control mouse and 5-HT^DR^-specific REV-ERBα cKO mouse using HPLC-ECD. **e** The amount of 5-HT in the NAc was quantified at CT00 and CT12 in control mice and 5-HT^DR^-specific REV-ERBα cKO mice using HPLC-ECD. In this and subsequent figures, all data are presented as the mean ± s.e.m. *n* = 4 for mRNA studies, and *n* = 3 for 5-HT level in DR and NAc, respectively. Statistical differences in control versus 5-HT^DR^-specific REV-ERBα cKO mice at two circadian time points were evaluated using two-way ANOVA followed by a post hoc comparison using Tukey’s test. ns, not significant, *P < 0.05, **P < 0.01, ***P < 0.001.

To study the functional role of REV-ERBα in DR 5-HTergic neurons, we first generated a 5-HT^DR^-specific REV-ERBα conditional knockout (cKO) mouse using the CRISPR/Cas9 gene-editing system by injecting an AAV vector with *Rev-erbα* single-guide RNA (sgRNA) into the DR of SERT (serotonin transporter, *Slc6a4*)-cre mice (Fig. 1a). The generation of the 5-HT^DR^-specific REV-ERBα cKO mouse model was confirmed by the loss of REV-ERBα in immunolabeled TPH2-positive neurons, a marker for 5-HT neurons, using immunohistochemistry (IHC) (Fig. 1b). We also calculated the REV-ERBα knockout efficiency by dividing the number of REV-ERBα negative neurons in TPH2 positive immunolabeled neurons over HA (hemagglutinin)^+^ and TPH2^+^ double-positive neurons, showing that most of the REV-ERBα immunoreactivity was absent (87.83 ± 1.15%; *n* = 12) in 5-HT neurons of REV-ERBα cKO mice (Supplementary Fig. 1).

We analyzed the mRNA expression profiles of *Tph2, Bmal1*, and several circadian clock genes in the DR at two circadian time points, CT00 and CT12 (Fig. 1c). The control mouse (SERT-cre) exhibited daily variations in *Tph2* and *Bmal1* mRNA expression with a high level at CT00 and a low level at CT12; however, daily variations in *Tph2* and *Bmal1* mRNA were not observed in 5-HT^DR^-specific REV-ERBα cKO mice with higher levels at both CT00 and CT12. Daily variations were also observed in circadian clock genes, *Per2* and *Cry2*, with high levels at CT12 and low levels at CT00 in the control mouse. However, their mRNA levels were slightly increased without clear daily variations in the REV-ERBα cKO. The control mice exhibited clear-cut circadian variation in *Rev-erbα* mRNA, and REV-ERBα cKO mice showed the lowest *Rev-erbα* mRNA levels at both CT points. Pet-1 is known as a transcription activator of Tph2 and a 5-HT neuronal marker, the expression of which is restricted to 5-HT neurons during neural development and adulthood^28^. Interestingly, no daily variation was observed in Pet-1 mRNA expression in the control mouse; however, slightly high levels were observed at both CT points in the REV-ERBα cKO condition (Fig. 1c).

The circadian pattern of 5-HT levels has been previously reported in rodents^11,12^. We confirmed the daily variation in 5-HT levels at two CT points using HPLC-ECD. The control mice exhibited daily variations in 5-HT levels in the DR and NAc with a high level at CT00 and a low level at CT12, while the 5-HT^DR^-specific REV-ERBα cKO mice showed no daily variations. Notably, 5-HT levels were sufficiently high in REV-ERBα cKO mice, particularly at CT12, suggesting that ablation of REV-ERBα in 5-HT^DR^ neurons may lead to a hyperserotonergic state (Fig. 1d).

### Ablation of 5-HT^DR^-specific REV-ERBα induced impairment of social behavior, especially in social preference, not social recognition

**Fig. 2:**
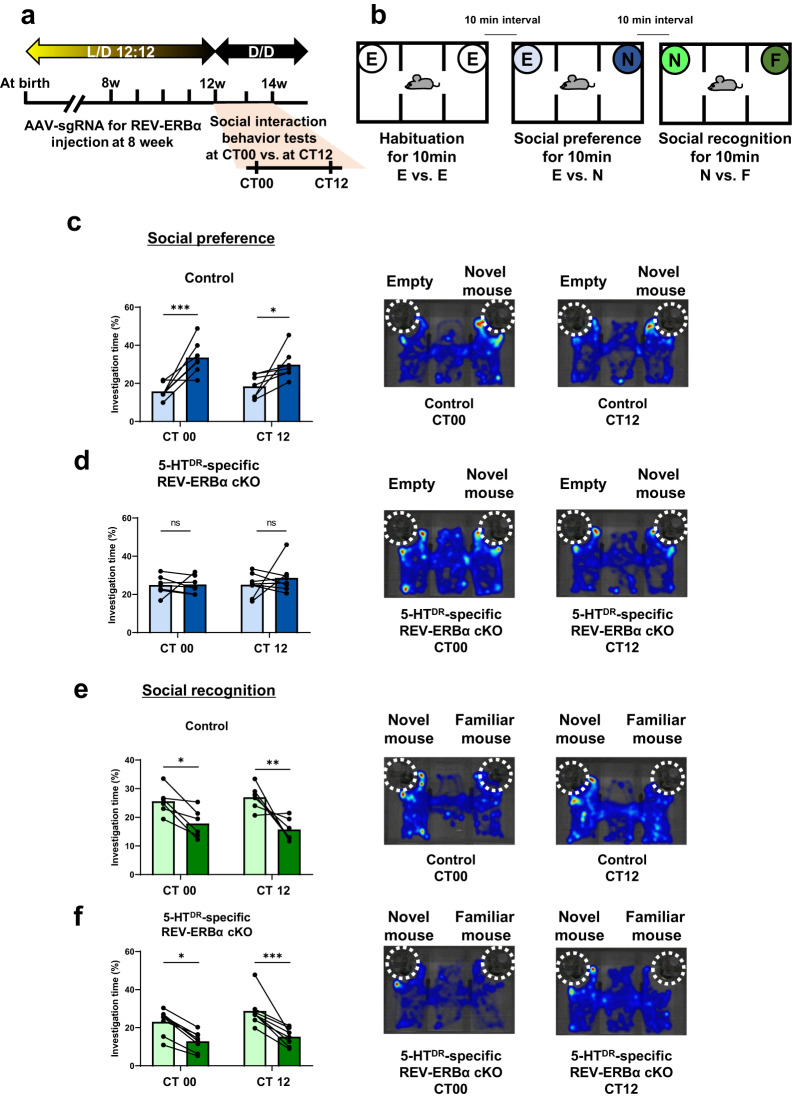
5-HT^DR^-specific REV-ERBα cKO mice exhibit impaired social interaction behavior, especially in social preference, but not social recognition, as revealed by the three-chamber behavior test. **a** Experimental schedule for AAV injection with Rev-erbα sgRNA in the DR of the SERT-cre mouse at 8 w and social interaction behavior test at two circadian time points, CT00 vs. CT12. **b** Experimental scheme of the three-chamber behavior test consisting of habituation, social preference, and social recognition tests. E, empty cup; N, novel mouse; F, familiar mouse **c** Quantification of the social preference test in control mice at CT00 and CT12. Note that the control mouse spent significantly more time interacting with a novel mouse than an empty cup at both CT00 and CT12. In this and subsequent figures, a representative heatmap image and dotted white circle indicate the location of the cup. **d** Quantification of the social preference test in 5-HT^DR^ REV-ERBα cKO mice at CT00 and CT12. Note that the DR 5-HT REV-ERBα cKO mice showed no difference in investigation time between the empty cup and the novel mouse at both CT00 and CT12. **e** Quantification of the social recognition test results in control mice at CT00 and CT12. Note that the control mice spent significantly more time interacting with the novel mouse than the familiar mouse at both CT00 and CT12. **f** Quantification of the social recognition test in 5-HT^DR^ REV-ERBα cKO mice at CT00 and CT12. Note that the 5-HT^DR^ REV-ERBα cKO mice spent significantly more time interacting with the cup containing the novel mouse than the cup containing the familiar mouse at both CT00 and CT12. Data are presented as the mean ± s.e.m. (*n* = 6–8). Statistical differences between an empty cup and novel mice (c and d) and between novel mice and familiar mice (e and f) at two circadian time points were evaluated using two-way ANOVA followed by a post hoc comparison using Tukey’s test. ns, not significant, *P < 0.05, **P < 0.01 ***P < 0.001.

To explore the possible impact of the ablation of REV-ERBα in 5-HT^DR^ neurons on social interaction behaviors, we examined social interaction behaviors with a three-chamber test at two CT time points following a recovery (~4 w) period after AAV microinjection into the DR (Fig. 2a). The three-chamber interaction assay consisted of three processes, habituation, social preference, and social recognition, with 10-min intervals between each test (Fig. 2b). In the habituation period with an empty cup in the right and left chambers, the mouse was allowed to explore the two chambers freely for 10 min. In the social preference test, a cup containing a novel mouse was placed in the right-side chamber, and an empty cup was placed in the left-side chamber. In the preference test, we quantified investigation time spent around the cup with a novel mouse vs. an empty cup. The control mice mostly investigated a cup with a novel mouse rather than the empty cup (Fig. 2c and Supplementary table), while 5-HT^DR^-specific REV-ERBα cKO mice did not show any difference in the investigation time between the two groups, exploring the novel mouse and empty cup for the same amount of time, indicating a deficit in the social preference behavior of REV-ERBα cKO mice (Fig. 2d and Supplementary table). In the social recognition test where another novel mouse was introduced into the empty cup on the left side, we quantified the difference in investigation time between the familiar and novel mouse for 10 min. Both control and 5-HT^DR^-specific REV-ERBα cKO mice spent a similar investigation time with the novel mouse compared to the familiar mouse regardless of the time points, CT00 or CT12 (Fig. 2e, f and Supplementary table). The total distance traveled during the three social behavior tests did not differ (data not shown). These results clearly indicate that the ablation of REV-ERBα in DR 5-HTergic neurons results in impaired social preference but not social recognition.

### Pharmacological manipulation of REV-ERBα through an agonist and/or antagonist affects social behaviors

Because several REV-ERBα agonists and/or antagonists are currently available and used in various experimental settings in vivo^29,30^, we hypothesized that the pharmacological manipulation of REV-ERBα activity can mimic social interaction behaviors, as shown in Fig. 2. First, using control mice, we examined whether pharmacological inhibition of REV-ERBα activity with SR8278, a REV-ERBα antagonist (16 µg/mouse) administered in the DR 3 hr before the behavioral test, may mimics the social interaction behaviors of 5-HT^DR^-specific REV-ERBα cKO mice. We compared the investigation time before (pretest) as a control and posttreatment of SR8278 to the DR in a three-chamber social interaction test.

**Fig. 3:**
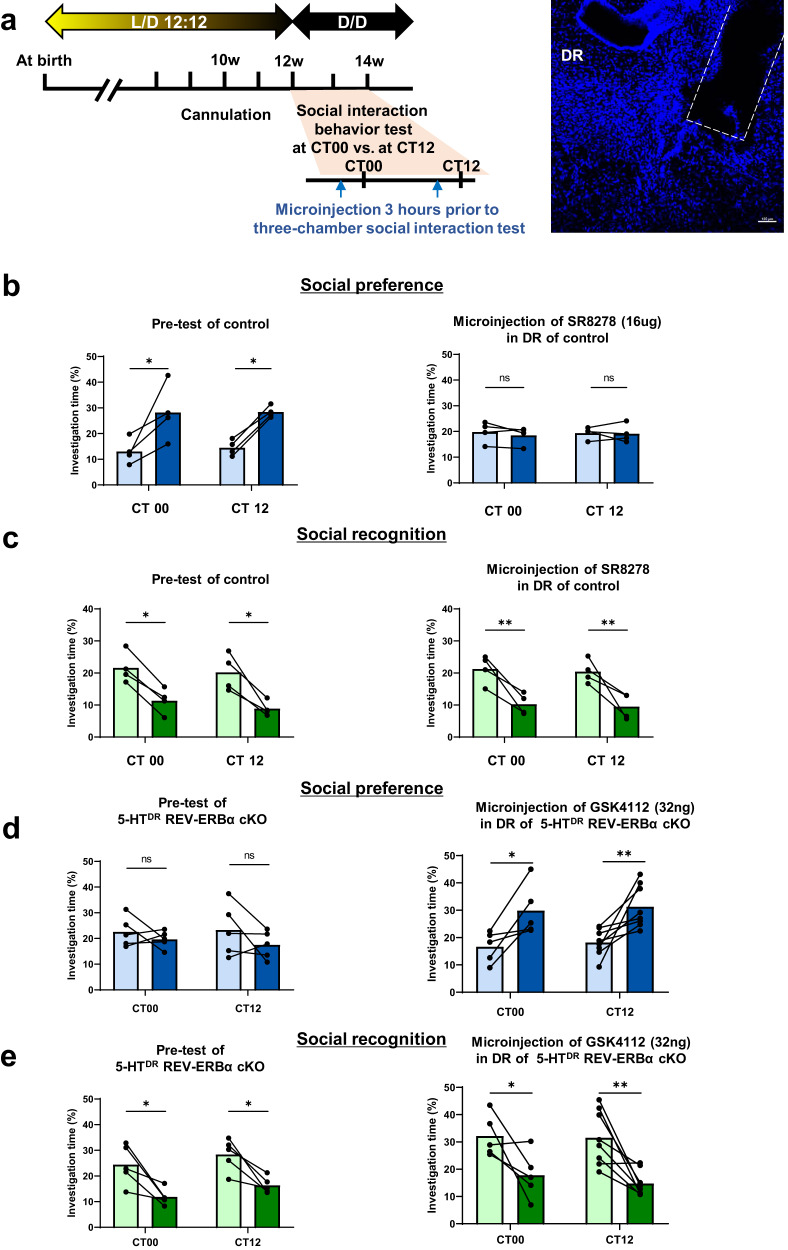
Pharmacological manipulation of REV-ERBα activity with its antagonist (SR8278) and agonist (GSK4112). **a** Experimental schedule for microinjection in the DR and social interaction behavior test at CT00 and CT12 and representative confocal image of the location of cannulation for antagonist or agonist treatment. **b** Quantification of the three-chamber social preference test in SR8278-treated control mice at CT00 and CT12. (left panel) The control mouse spent significantly more time interacting with the novel mouse than the empty cup at both CT00 and CT12 in the pretest. (right panel) Microinjection of SR8278-treated control mice exhibited decreased investigation time with the novel mouse at both CT00 and CT12. **c** Quantification of the social recognition test in SR8278-treated control mice at CT00 and CT12. The control mice with pre- and posttreatment of SR8278 spent significantly more time interacting with the cup with a novel mouse than the cup with the familiar mouse at both CT00 and CT12. **d** Quantification of the social preference test in GSK4112-treated 5-HT^DR^ REV-ERBα cKO mice at CT00 and CT12. The mice in the pretest spent significantly less time interacting with the cup containing the novel mouse than the empty cup at both CT00 and CT12. Mice receiving microinjection of GSK4112 interacted more with the novel mouse than the pretest mice at both CT00 and CT12. **e** Quantification of the three-chamber social recognition test in GSK4112-treated 5-HT^DR^ REV-ERBα cKO mice at CT00 and CT12. The WT mice pre- and posttreatment with GSK4112 spent significantly more time interacting with the cup containing the novel mouse than with the cup containing the familiar mouse at both CT00 and CT12. Data are presented as the mean ± s.e.m. (*n* = 4–8). Statistical differences between an empty cup and novel mouse (**b**, **d**) and between a novel mouse and familiar mouse (c and d) at two circadian time points were evaluated using two-way ANOVA followed by a post hoc comparison using Tukey’s test. ns, not significant, *P < 0.05, **P < 0.01.

During the pretest period, control mice investigated a wire cup with a novel mouse longer than an empty cup in the social preference test and spent more investigation time with the novel mouse than the familiar mouse in the social recognition test. Interestingly, the control mouse with microinjection of SR8278 into the DR showed no social preference to investigate novel mice (Fig. 3b and Supplementary table), confirming the previous finding shown in the REV-ERBα cKO mice (Fig. 2b). There was no difference in social preference at the two CT points. The social recognition test revealed no alterations in social recognition between the pre- and postperiod with no difference between the two CT points (Fig. 3c and Supplementary table).

Conversely, we tested whether the impaired social preference revealed in DR 5-HT REV-ERBα cKO mice (Fig. 2d and pretest in Fig. 3d) could be restored by microinjection of GSH4112, a potent REV-ERBα agonist, in the DR 3 h before the behavioral tests. Interestingly, in the social preference test, administration of GSK4112 (32 ng) induced social preference only at CT00 and CT12. (Fig. 3d, right and Supplementary table). There was no effect on social recognition owing to a similar investigation time during the pre- and posttreatment of GSK4112 (Fig. 3e and Supplementary table). These results indicate that the pharmacological manipulation of REV-ERBα activity with its agonist and/or antagonist partially mimicked social interaction behaviors and restored deficits in the control and 5-HT^DR^-specific REV-ERBα cKO mice.

### Changes in real-time circadian 5-HT neuron activity together with increased 5-HT levels by fiber photometry during the social preference test

**Fig. 4:**
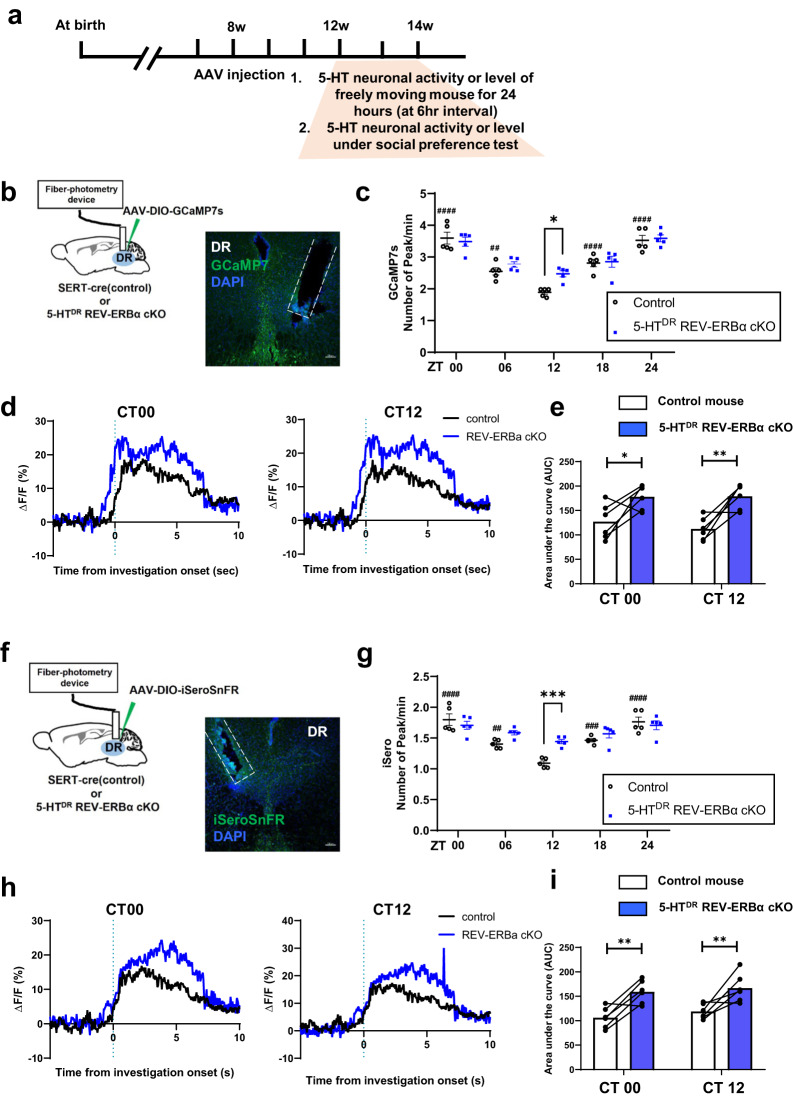
5-HT^DR^-specific REV-ERBα cKO mice show high neural activity in response to GCaMP7s and real-time 5-HT levels in the DR determined by iSeroSnFR. **a** Experimental schedule for fiber photometry for the measurement of GCaMP7s and iSeroSnFR activity in control and 5-HTDR-specific REV-ERBα cKO mice at 6-h intervals per day (24 h). **b** Schematic of stereotactic injection of AAV-mediated GCaMP7s and optic fibers in the DR of control and 5-HT^DR^-specific REV-ERBα cKO mice by fiber photometry recording and representative image of GCaMP7s expression and fiber location. **c** Freely moving 5-HT^DR^-specific REV-ERBα cKO mice exhibited significantly increased neural activity at CT12 compared to control mice. The peak numbers of GCaMP7s fluorescence activity per min. **d**, **e** Ca^2+^ signals of 5-HT^DR^-specific REV-ERBα cKO mice during the social preference test were increased both at CT00 (left) and CT12 (middle), as revealed by the area under the curve (AUC) analysis of each mouse (right). **f** Schematic of stereotactic injection of iSeroSnFR and implantation of optic fiber in the DR of control and 5-HT^DR^-specific REV-ERBα cKO mice for in vivo fiber photometry recording and representative image of GCaMP7s expression and fiber location. **g** Freely moving 5-HT^DR^-specific REV-ERBα cKO mice exhibited significantly increased 5-HT levels at CT12 compared to control mice, as determined by the peak fluorescence activity per min. **h**, **i** The 5-HT levels of 5-HT^DR^-specific REV-ERBα cKO mice during the social preference test were increased at both CT00 (left) and CT12 (middle), as revealed by AUC analysis of each mouse (right). Data are presented as the mean ± s.e.m. (*n* = 5 for (**c** and **g**), and *n* = 6 for **d**, **e**, **h**, and **i**). Statistical differences in groups were evaluated using two-way ANOVA followed by a post hoc comparison using Sidak’s test for the number of peaks and Tukey’s test for AUC analysis. *P < 0.05, **P < 0.01, ***P < 0.001 for control versus cKO and ^##^*P* < 0.01, ^####^*P* < 0.0001 for comparing each time point to the lowest value in control.

To explore whether the ablation of REV-ERBα alters 5-HT neuron activity in the DR, we measured 5-HT neuron activity with an imaging sensor, GCMP7s, by fiber photometry. Initially, we determined the circadian profiles of 5-HT neuron activity for 60 mins during five zeitgeber time (ZT) time intervals using an in vivo fiber-photometry recording device. A fiber optic cannula was implanted above the DR of each animal, and the neuron activity was quantified by the number of peaks per min (Fig. 4a, b). The control mice exhibited circadian variations in DR 5-HT neuron activity with the highest level at ZT00 and lowest level at ZT12, whereas the REV-ERBα cKO mice showed similar variations in 5-HT neuron activity, except at CT12, where the neuron activity was statistically increased compared to that of the control mice (Fig. 4c). Then, we tested whether 5-HT neuron activity may be altered during the social preference test in control and REV-ERBα cKO mice. We analyzed the real-time dynamics of GCaMP7s within a short time when the subject mouse simply interacted with a novel mouse during a social preference test. Statistical analysis with the ∆F/F index showed that the total area of neuron activity summed in the REV-ERBα cKO mice was broader than that of the control mice at CT00 and CT12 (Fig. 4d, e).

We also measured the 5-HT tone in the DR using an imaging sensor, iSeroSnFR, which is a fluorescence protein-based biosensor detecting extracellular 5-HT release (Unger et al., 2020). Similar to fiber photometry with GCaMP7s, real-time 5-HT dynamics were measured for 1 h at 6-h intervals one day. We recorded 5-HT dynamics in the DR of control and REV-ERBα cKO mice and calculated the number of peaks per min in a day. Circadian variation in extracellular 5-HT tones in REV-ERBα cKO mice was high at ZT12 compared with that in the control group (Fig. 4g). Increased 5-HT tone activity in DR 5-HT neurons in REV-ERBα cKO mice was similar to the shapes determined by GCaMP7 shown in Fig. 4d, e. In the social preference test, the real-time dynamics of 5-HT tones within a short time domain when the subject mouse interacted with a novel mouse in the social preference test were quite similar to those of Ca^2+^ signals (Fig. 4g, i).

**Fig. 5:**
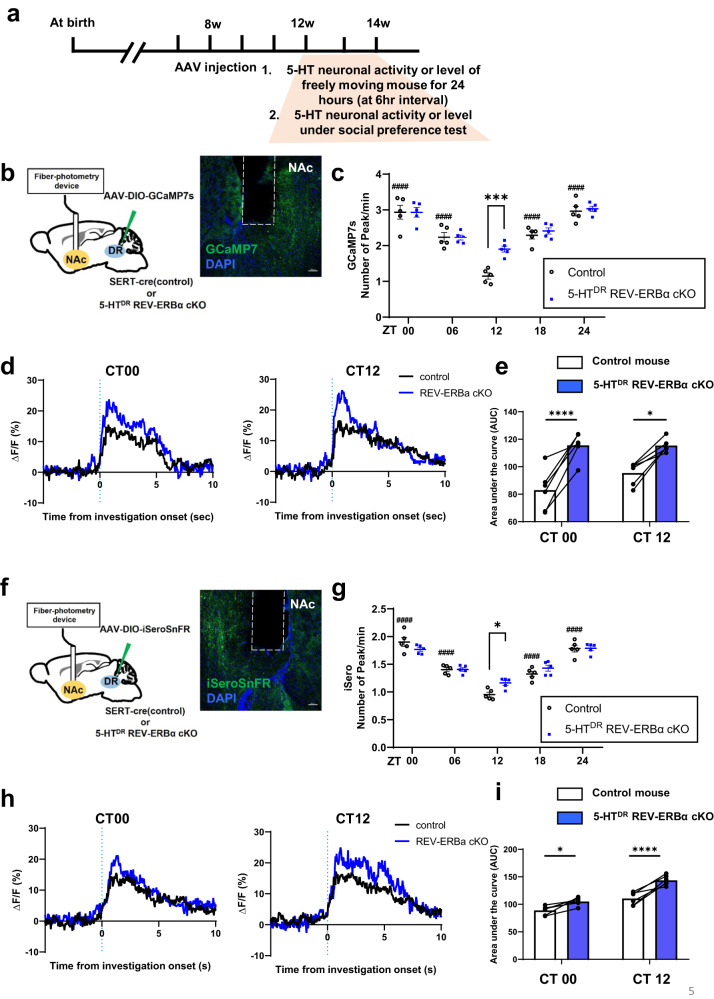
5-HT^DR^-specific REV-ERBα cKO mice show high neural activity in response to GCaMP7s and real-time 5-HT levels in the NAc. **a** Experimental schedule for fiber photometry for the measurement of GCaMP7s and iSeroSnFR activity in control and 5-HT^DR^-specific REV-ERBα cKO mice at 6-h intervals per day (24 h). **b** Schematic of stereotactic injection of AAV-mediated GCaMP7s and optic fibers in the NAc of control and 5-HT^DR^-specific REV-ERBα cKO mice by fiber photometry recording. **c** Freely moving 5-HT^DR^-specific REV-ERBα cKO mice showed significantly increased neural activity at CT12 in the NAc compared to control mice. The peak numbers of GCaMP7s fluorescence activity per min. **d**, **e** Ca^2+^ signals of 5-HT^DR^-specific REV-ERBα cKO mice during interaction with a novel mouse on the social preference test were increased both at CT00 (left) and CT12 (middle), revealed by AUC analysis of each mouse (right). **f** Schematic of stereotactic injection of iSeroSnFR and implantation of optic fibers in the NAc of control and 5-HT^DR^-specific REV-ERBα cKO mice for in vivo fiber photometry recording **g** Freely moving 5-HT^DR^-specific REV-ERBα cKO mice exhibited significantly increased 5-HT levels at CT12 compared to control mice, as determined by the peak fluorescence activity per min. **h**, **i** The 5-HT levels of 5-HT^DR^-specific REV-ERBα cKO mice during the social preference test were increased at both CT00 (left) and CT12 (middle), as revealed by AUC analysis of each mouse (right). Data are presented as the mean ± s.e.m. (*n* = 5 for (**c** and **g**), and *n* = 6 for **d**, **e**, **h**, and **i**). Statistical differences in groups were evaluated using two-way ANOVA followed by a post hoc comparison using Sidak’s test for the analysis of the number of peaks and Tukey’s test for AUC analysis. *P < 0.05, ***P < 0.001, ****P < 0.0001 for control versus cKO and ^####^*P* < 0.0001 for each time point compared to the lowest value in control.

To test whether the neural activity or 5-HT tone changes in the target region, we inserted the optic cannula into the NAc in each of the control and 5-HT^DR^-specific REV-ERBα cKO mice and measured the profiles of 5-HT neuron activity with GCMP7s by fiber photometry (Fig. 5a, b); the neuron activity was quantified by the number of peaks per min. The circadian variation of 5-HT neurons in the DR projecting into the NAc in the control mice exhibited the highest level at ZT00 and lowest level at ZT12, whereas the REV-ERBα cKO mice had similar variations in 5-HT neuron activity; however, the neuron activity was statistically increased compared to that of the control mice at CT12 similar to the DR region (Fig. 5c). Then, we tested whether 5-HT neuronal activity is altered during the social preference test in the NAc of control and REV-ERBα cKO mice. Although there was no difference at the two time points, AUC analysis of ∆F/F during social preference in REV-ERB cKO mice was also increased compared with that in control mice (Fig. 5d, e).

When measuring 5-HT tones in the NAc using an iSeroSnFR, circadian variation in NAc 5-HT levels in REV-ERBα cKO mice was high only at ZT12 compared with the control group (Fig. 5g). In the social preference test, the real-time dynamics of 5-HT tones in the REV-ERBα cKO mice when the subject mouse interacted with a novel mouse in the social preference test were higher than those of the control mice (Fig. 5h, i).

### DR-NAc circuit-specific ablation of REV-ERBα in 5-HT neurons resulted in a deficit in social preference

**Fig. 6:**
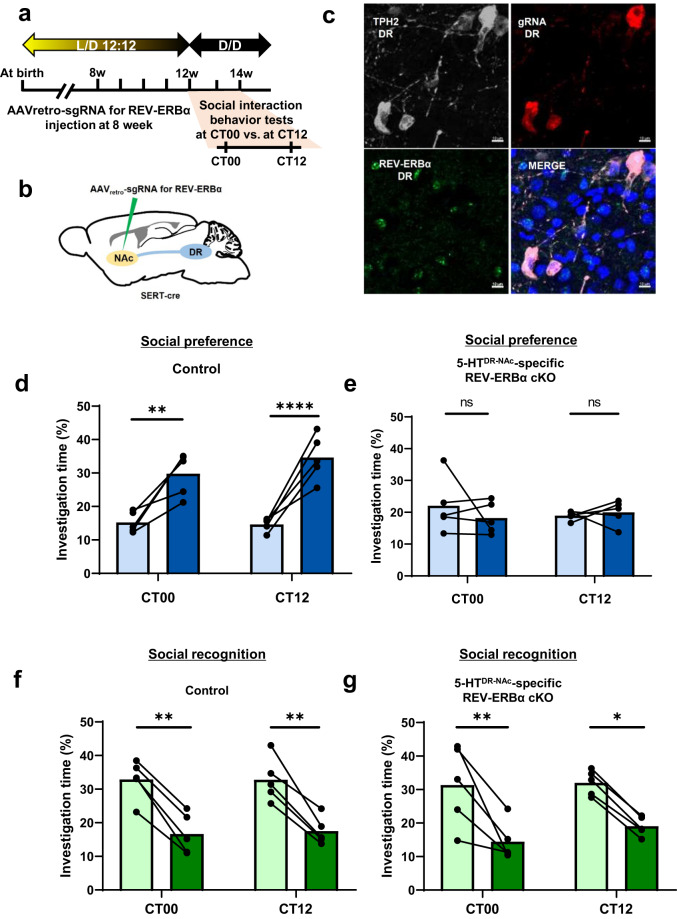
DR-NAc circuit-specific ablation of REV-ERBα in 5-HT neurons resulted in deficits in social preference. **a** Experimental schedule for AAV_retro_ injection at 8 w and social interaction behavior test performed at two circadian time points, CT00 vs. CT12. **b** Schematic of stereotactic injection of Rev-erbα sgRNA in the NAc of SERT-cre mice. **c** Representative image of the DR region after 4 weeks of recovery of AAV_retro_-sgRNA injection. **d** Quantification of the social preference test in a control mouse. The control mouse spent significantly more time interacting with the novel mouse than the empty cup at both CT00 and CT12. **e** Quantification of the social preference test in 5-HT^DR-NAc^-specific REV-ERBα cKO mice. 5- HT^DR-NAc^ REV-ERBα cKO mice also spent less time interacting with the novel mouse at both CT00 and CT12. **f** Quantification of the social recognition test in control mice at CT00 and CT12. The control mice spent significantly more time interacting with the novel mouse than the familiar mouse at both CT00 and CT12. **g** Quantification of the social recognition test in 5-HT^DR-NAc^ REV-ERBα cKO mice at CT00 and CT12. Notably, the 5-HT^DR-NAc^ REV-ERBα cKO mice spent significantly more time interacting with the novel mouse than the familiar mouse at both CT00 and CT12. (*n* = 5). Data are presented as the mean ± s.e.m. (*n* = 5). Statistical differences between an empty cup and novel mouse (**c**, **d**) and between a novel mouse and familiar mouse (**e**, **f**) at two circadian time points were evaluated using two-way ANOVA followed by a post hoc comparison using Tukey’s test. ns, not significant, *P < 0.05, **P < 0.01, ****P < 0.0001.

We next investigated how social preference changes when Rev-erbα was specifically eliminated from 5-HTergic neurons in the DR-NAc circuit. Briefly, we generated 5-HT^DR-NAc^-specific Rev-erbα cKO in combination with AAV_retro_-sgRNA for Rev-erbα in SERT-cre; Rosa26-LSL-Cas9-expressing mice. After 4 weeks of recovery, we performed the three-chamber social interaction test and quantified the investigation time (Fig. 6a, b). We also calculated the REV-ERBα knockout efficiency by dividing the number of REV-ERBα negative neurons among TPH2 positive immunolabeled neurons over the number of mCherry and TPH2 double-positive neurons. Most of the REV-ERBα^+^ immunoreactivity was absent (87.45 ± 0.98%; *n* = 10) in the 5-HT neurons of REV-ERBα knockout mice (Fig. 6c and Supplementary Fig. 2).

In the social preference test, although there was no difference in the investigation between two time points, CT00 and CT12, the control mice investigated a cup containing a novel mouse rather than an empty cup (Fig. 6d), while 5-HT^DR-NAc^-specific Rev-erbα cKO mice did not show any difference in the investigation time between a cup with a novel mouse and an empty cup (Fig. 6e). However, as with 5-HT^DR^-specific Rev-erbα cKO mice, both control and 5-HT^DR-NAc^-specific Rev-erbα cKO mice spent a similar investigation time with the novel mouse compared to the familiar mouse, with no difference between the two time points, CT00 and CT 12, in the social recognition test (Fig. 6f, g). This result suggests that the abnormal social preference induced by ablation of Rev-erbα in DR 5-HT neurons is specific to the DR-NAc circuit.

### Optogenetic inhibition of DR 5-HTergic projections to the NAc rescued impaired social preference in REV-ERBα cKO mice

**Fig. 7:**
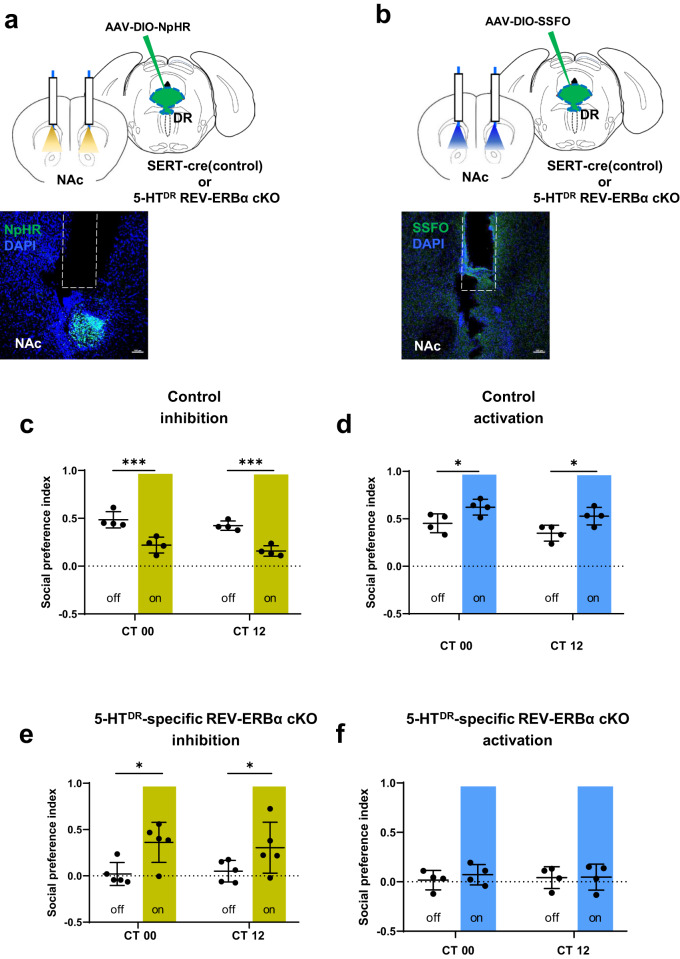
Optogenetic inhibition of DR 5-HTergic projections into the NAc rescued impaired social preference in DR 5-HT REV-ERBα cKO mice. **a** Schematic of AAV-DIO-NpHR and bilateral optic fiber implantation targeting the NAc. **b** Schematic of AAV-DIO-SSFO and bilateral optic fiber implantation targeting the NAc. **c**. Optogenetic inhibition of DR 5-HT in the NAc circuit in control mice significantly impaired social preference, as revealed by a decrease in the social preference index. **d** Optogenetic activation of DR 5-HT in the NAc circuit in control mice significantly enhanced social preference, as revealed by an increase in the social preference index. **e** Optogenetic inhibition of DR 5-HT in the NAc circuit in REV-ERBα cKO mice significantly rescued the impairment of social preference, as revealed by an increase in the social preference index. **f** Optogenetic activation of the DR 5-HT-to-NAc circuit in REV-ERBα cKO mice was not different before activation and after activation. Data are presented as the mean ± s.e.m. (*n* = 4). Statistical differences in groups were evaluated using two-way ANOVA followed by a post hoc comparison using Sidak’s test between lights off and lights on at each time point. n.s., not significant, *P < 0.05, ***P < 0.001.

Next, we examined whether optogenetic modulation of the DR-NAc 5-HTergic circuit may rescue abnormal social preference. We injected AAV expressing stabilized step function opsin (SSFO) for optogenetic activation (Yizhar et al., Nature, 2011) or NpHR for optogenetic inhibition (Fig. 7a, b).

Bilateral optogenetic activation of the DR to NAc 5-HTergic circuit increased social preference, while inhibition of this circuit decreased social preference in control mice (Fig. 7c, d and Supplementary table). Notably, bilateral optogenetic inhibition of the DR-NAc 5-HTergic circuit in REV-ERBα cKO mice led to increased social preference, whereas optogenetic activation resulted in no change in social preference (Fig. 7e, f and Supplementary table). These results suggest that social preference is influenced by REV-ERBα in DR 5-HT neurons, specifically in a DR-NAc circuit-dependent manner.

## Discussion

The present study demonstrated for the first time that the circadian nuclear receptor REV-ERBα affected social interaction behaviors, particularly inducing impairment of social preference but not social recognition in 5-HT^DR^-specific REV-ERBα cKO mice. Our major findings are as follows: (1) Circadian regulation of 5-HT neurons appears to be under circadian timing, resulting in daily alterations in mRNA expression of 5-HT-related and clock genes along with changes in 5-HT levels in the DR. (2) Genetic ablation of 5-HT^DR^-specific REV-ERBα cKO induced a deficit in social preference but not social recognition behavior, which can be mimicked by pharmacological manipulation of REV-ERBα activity with its potent antagonist and rescued by treatment with a REV-ERBα agonist. (3) Social preference is functionally associated with alterations in real-time 5-HT neuron activity along with 5-HT levels determined by fiber photometry, and (4) optogenetic inhibition of 5-HTergic projections from the DR to the NAc restored impaired social preference in 5-HT^DR^-specific REV-ERBα cKO mice.

As mentioned above, 5-HT neurons in the DR are influenced by the control of the circadian timing system, where the central master oscillator is located in the suprachiasmatic nucleus (SCN) in the anterior hypothalamus. We found that the mRNA expression of 5-HT-related genes, such as *Tph2* and several clock genes, is altered daily along with changes in 5-HT levels in the DR in control mice, and the tissue-specific genetic knockout of *Rev-Erbα* profoundly affects the circadian rhythmicity of 5-HT neurons in the DR, as shown in Fig. 1. *Pet-1* mRNA expression was slightly upregulated without a daily oscillation at two circadian time points when the *Rev-Erbα* gene was knocked out. Although *Pet-1* is a 5-HT neuronal marker, the expression of which is restricted to 5-HT neuron-specific development^28^, its physiological function during adulthood is largely unknown and should be studied in the future. Notably, 5-HT levels were sufficiently high in the DR and NAc of 5-HT^DR^-specific REV-ERBα cKO mice, particularly at CT12, suggesting that the genetic ablation of REV-ERBα, which acts as a transcriptional repressor of the Tph2 gene machinery of 5-HT biosynthesis, may result in a hyperserotonergic state. This hyperserotonergic level at CT12 appears to be well associated with the circadian pattern of 5-HT neuron activity as well as 5-HT levels in the DR and NAc (Figs. 4, 5).

One of the interesting findings is that there is no clear difference in social interaction behavioral patterns when examined at two circadian time points, CT00 and CT12. In fact, there was no difference between the social behavioral index (an investigation time) of the two CT points, indicating that social interaction behaviors might occur in a circadian rhythm-independent manner. The circadian fluctuations in mRNA expression of 5-HT-related genes and clock genes along with 5-HT levels complicate the proposal of a suitable explanation for unexpected results in this study. However, Yang et al. (2008)^31^ showed the presence of sociability using a three-chamber sociability test; for instance, a mouse showed greater interest in interacting with a novel mouse, regardless of the light or dark conditions. However, further study, such as a more detailed time course experiment, is needed in the near future.

REV-ERBα is a potent nuclear receptor that plays an important role in transcriptional repression modulating *Bmal1* gene expression by competitive binding with ROR*α* in the stabilizing loop of the circadian time-keeping system^14^. We also previously found that REV-ERBα, as a transcription repressor, regulates tyrosine hydroxylase (*TH*) gene transcription via competition with NURR1, another nuclear receptor-related 1 protein in midbrain DAergic neurons, thereby driving circadian oscillation of *TH* gene expression^15^. The question of whether the molecular action of REV-ERBα in 5-HTergic neurons is similar to that shown in DAergic neurons is important to address. The present study evidently showed that pharmacological manipulation of REV-ERBα activity with its potent agonist and/or antagonist successfully affected social interaction behaviors (Fig. 3), indicating that synthetic REV-ERBs (REV-ERBα/β) ligands would be beneficial in the treatment of social behavior-related disorders. Indeed, increasing evidence suggests that REV-ERBs (REV-ERBα/β) are heavily involved in numerous physiological functions, including hepatic lipid and glucose metabolism, skeletal muscle oxidative capacity, adipogenesis, and inflammatory response^32,33^. The possible involvement of ‘non-clock genes’ in social interaction behaviors can therefore be presumed. Moreover, the putative non-clock gene may have an authentic responsive element (RORE) of the *Rev-Erb*α gene.

Studies show that 5-HT neurons account for two-thirds of the total neurons in the DR. 5-HT neurons receive broad and dense inputs from a broad range of forebrain and limbic structures that are significantly involved in social and emotional behavior^34^. Moreover, 5-HT neurons are released throughout the brain to trigger a wide range of signaling pathways via at least 14 receptors^35^. 5-HT neurons in the DR are heterogeneous in cell morphology, neurochemical markers, functional topography, and electrophysiological properties^36,37^. However, the DR is biochemically and anatomically diverse and heterogeneous and contains some other neurotransmitters, such as DA, glutamate, and GABA^38,39^. Recently, Matthews et al. (2016)^40^ revealed that DA neurons in the DR may be involved in the regulation of social interaction. However, because the local circuit between 5-HT and DA or other neurons within the DR has not been fully studied, more in-depth research about the DR local circuit is needed to determine the precise mechanism of sociability regulated by DR. The increased levels of 5-HT at CT12 are associated with a deficit in social preference, suggesting that the circadian rhythm of 5-HT homeostasis may play an important role in regulating social interaction behaviors. Notably, DR is anatomically segregated and functionally distinct in circuit connectivity based on the genetic dissection of conditional Tph2 KO and circuit-based functional studies using a chemogenetic approach^3^.

The present finding that AAV-mediated genetic ablation of DR 5-HT neuron-specific REV-ERBα cKO induced impairment of social behaviors, particularly in social preference, but not social recognition appears to be quite important. Therefore, based on the above notion^3^, we attempt to explain the neural circuit and underlying mechanism involved in social interaction behaviors, particularly social preference behavior. Recently, Walsh et al. (2018)^6^ found that modulation of 5-HT release from the DR to the NAc bidirectionally modified sociability in an ASD animal model, where genetic deletion of the syntenic region from the 5-HT neurons induced a deficit in social preference behavior and decreased DR 5-HT neuron activity. Optogenetic activation of DR 5-HT neurons rescued deficits in social preference behavior, and pharmacological activation of the 5-HT1b receptor in the NAC evidently enhanced sociability. However, social recognition is based on social memory, namely, the ability to recognize and remember familiar conspecifics. The hippocampal dorsal CA2 and dorsal CA1 subregions are critical for social memory^41,42^ and memory storage^43,44^. The medial septal region was recently identified as an extrahippocampal input to the dorsal CA2 subregion^7^. This medial septal region appears to be important for social memory formation, thereby affecting memory stability. Moreover, the optogenetic manipulation of median raphe (MR) 5-HT nerve terminals, probably through the 5-HT1b autoreceptor, in the medial septum bidirectionally regulates social memory stability. The two different neural circuits are involved in social preference vs. social recognition behaviors, which may explain the impact of REV-ERBα cKO on the DR to NAc circuit for social preference behavior.

Abnormal social preference is one of the critical features of autism spectrum disorder (ASD)^45^. Although hyperserotonemia, where 5-HT levels were increased in blood and platelets, was observed in patients with ASD^46^, how hyperserotonemia induces a deficit of social preference remains unexplored. Recent studies have demonstrated that hyperserotonemia in the brain is closely related to a deficit in social preference and can be alleviated by tryptophan depletion in serotonin transporter (SERT) mutant and KO animal models^9,47^. Similarly, our results showed that 5-HT levels in cKO mice increased significantly at CT12 in the DR and in the target region, the NAc.

In summary, based on an AAV-mediated genetic animal model and dissection of circuit wiring using novel tools, our study demonstrated that the circadian nuclear receptor REV-ERBα in DR affects social preference behaviors. Then, our discoveries may elucidate the neural mechanisms of social interaction behaviors and offer additional insight into novel clinical approaches toward treating neurological diseases, notably, ASD.

## Supplementary information

supplementary figure

supplementary table
