# Supplementary material for: Impact of the circadian nuclear receptor REV-ERBα in dorsal raphe 5-HT neurons on social interaction behavior, especially social preference
Source: Exp Mol Med. 2023 Aug 3;55(8):1806–19. doi: 10.1038/s12276-023-01052-7 (PMC10474013; doi:10.1038/s12276-023-01052-7)
Supplement: Supplementary file 1 — supplementary figure [file 12276_2023_1052_MOESM1_ESM.pdf]

Supplementary information for

‘Impact of the circadian nuclear receptor REV-ERBα in dorsal raphe 5-HT neurons on social interaction behavior, especially social preference’

Supplementary Figure 1

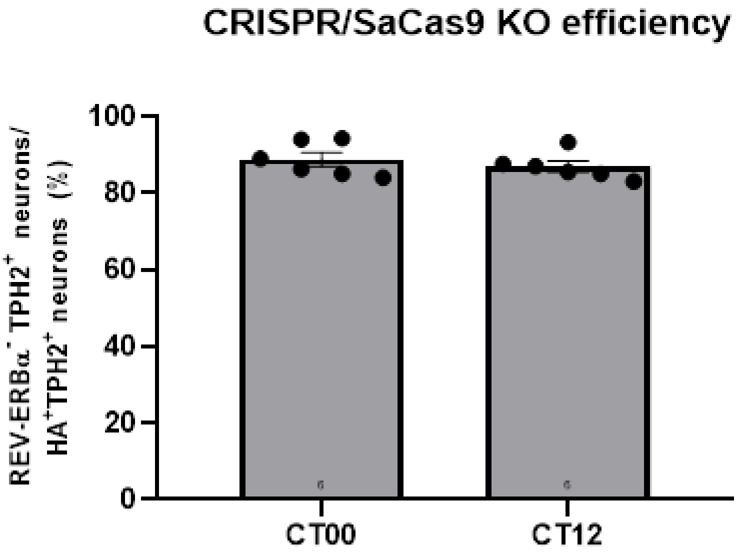

Supplementary Fig. 1. Loss of immunolabelled REV-ERBα in DR 5-HT neurons in DR 5-HT REV-ERBα cKO. Quantification of CRISPR/SaCas9 efficiency by AAV-sgRNA in the dorsal raphe of 5-HT^{DR} REV-ERBα cKO at CT00 and CT12. KO efficiency was calculated by dividing the REV-ERBα^{-} negative neurons in TPH2 positive neurons by HA^{+} and $TPH2^{+}$ double-positive neurons. Data are represented as mean ± s.e.m. (CT00: 88.77 ± 1.84 and CT12: 86.90 ± 1.44)

Supplementary Figure 2

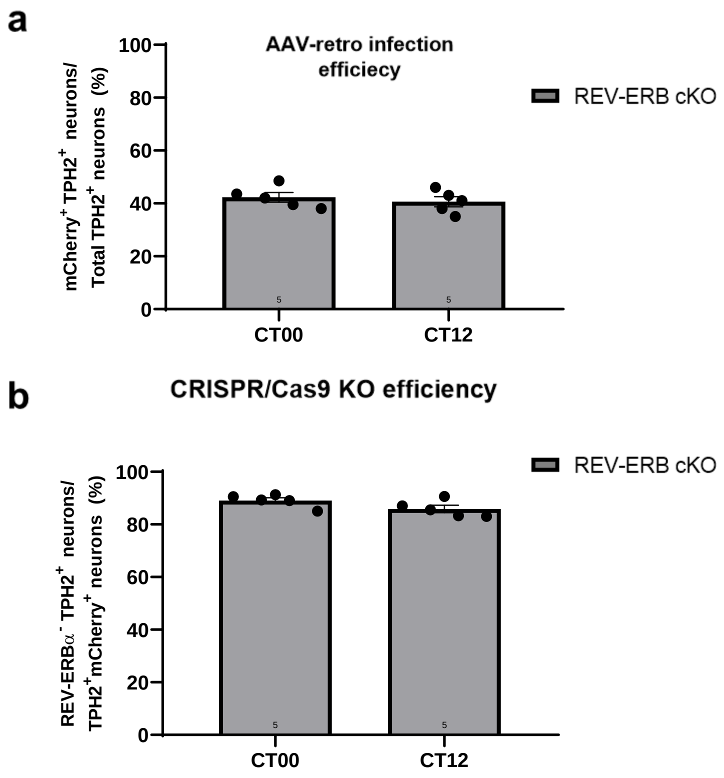

Supplementary Fig. 2. DR-NAc circuit-specific ablation of REV-ERBα in 5-HT decreased the number of immunolabelled REV-ERBα in DR 5-HT neurons.

a. Quantification of retrograde CRISPR/Cas9 efficiency by AAVretro-sgRNA at CT00 and CT12. The efficiency of AAV-retro sgRNA was calculated by dividing mCherry and TPH2 double positive neurons over TPH2 positive neurons. Data are represented as mean ± s.e.m. (CT00: 42.3 ± 1.82 and CT12: 40.6 ± 1.91) **b.** Quantification of CRISPR/Cas9 efficiency by AAVretro-sgRNA at CT00 and CT12. KO efficiency was calculated by dividing the REV-ERBα^{-} negative neurons in TPH2^{+} positive neurons over mCherry^{+} and TPH2^{+} double-positive neurons. Data are represented as mean ± s.e.m. (CT00: 89.02 ± 1.09 and CT12: 85.88 ± 1.39)
